# A permanent host shift of rabies virus from *Chiroptera* to *Carnivora* associated with recombination

**DOI:** 10.1038/s41598-017-00395-2

**Published:** 2017-03-21

**Authors:** Nai-Zheng Ding, Dong-Shuai Xu, Yuan-Yuan Sun, Hong-Bin He, Cheng-Qiang He

**Affiliations:** Shandong Provincial Key Laboratory of Animal Resistance Biology, College of Life Science, Shandong Normal University, Jinan, 250014 China

## Abstract

Bat virus host shifts can result in the spread of diseases with significant effects. The rabies virus (RABV) is able to infect almost all mammals and is therefore a useful model for the study of host shift mechanisms. Carnivore RABVs originated from two historical host shifts from bat viruses. To reveal the genetic pathways by which bat RABVs changed their host tropism from bats to carnivores, we investigated the second permanent bat-to-carnivore shift resulting in two carnivore variants, known as raccoon RABV (RRV) and south-central skunk RABV (SCSKV). We found that their glycoprotein (G) genes are the result of recombination between an American bat virus and a carnivore virus. This recombination allowed the bat RABV to acquire the head of the G-protein ectodomain of the carnivore virus. This region is involved in receptor recognition and binding, response to changes in the pH microenvironment, trimerization of G proteins, and cell-to-cell transmission during the viral infection. Therefore, this recombination event may have significantly improved the variant’s adaptability to carnivores, altering its host tropism and thus leading to large-scale epidemics in striped skunk and raccoon.

## Introduction

Bat virus host shifts have resulted in the emergence of several serious diseases in humans and animals, such as SARS^1^, Ebola^2^, and rabies^3^ viruses. To predict and control the outcome of these host shifts, it is crucial to determine the genetic pathways by which these viruses rapidly adapt new host species, which are poorly understood for most host–virus systems^4^.

The rabies virus (RABV; genus *Lyssavirus*, Family *Rhabdoviridae*) is one of the most significant pathogens threatening animal and human health, exhibiting a high fatality rate. This virus has the capacity to infect almost all mammals. Although RABV is usually maintained in distinct host species-associated transmission cycles, typically within the *Carnivora* and *Chiroptera* ^5–7^, host shift events are still of public health concern due to increasing risks of human exposures^7^. Recent host shifts between carnivores include the re-emergence of RABV in wild Taiwan ferret-badgers^8^, as well as the outbreaks of domestic dog-associated RABV in Ethiopian wolves^9^ and of striped skunk-associated RABV in gray foxes in California^10^. It has also been reported that multiple host shifts between bat and mesocarnivores have occurred with the big brown bat-associated RABV in Arizona^11^. In South America, the vampire bat, a reservoir of RABV, is resposible for the high number of rabies cases among terrestrial mammals in the rural cycle^12^. Therefore, RABV is a useful model for studying the mechanisms of bat virus host shifts^13^.

Multiple factors may be involved in RABV host shifts, including viral adaptability, host barriers, ecological factors, and human interventions^7^. Of these, viral adaptability may play a significant role. Interestingly, although evolutionary analyses have demonstrated that bat-to-carnivore host-shifting viruses accumulate few adaptive mutations^4, 6, 11^, in none of these examples did bat RABV establish permanent transmission cycles in the new host species^7^.

To date, there have been two permanent host shifts from the *Chiroptera* to the *Carnivora* in the history of lyssaviruses, with disastrous results^6^. The first occurred thousands of years ago and has resulted in worldwide rabies epidemics in terrestrial mammals through the present^6^. The second permanent host shift appears to have occurred in North America, producing a lineage including two variants, raccoon rabies virus (RRV) and south-central skunk variant (SCSKV). SCSKV mainly circulates in striped skunks and sporadically in other terrestrial animals, such as wolf. The RRV variant was first found in raccoons in Northern America and has seriously threatened the health of raccoons^6, 14^. Before the 1970s, rabid raccoons were limited to the southeastern United States^15^. Following the detection of RRV in West Virginia in the 1970s, however, the distribution of rabid raccoons has spread at an alarming rate, becoming an epizootic that affects many thousands of square kilometers in North America^15, 16^. Until 1998, raccoons have had the highest incidence of rabies of all species in the United States^17^. Elucidating the genetic mechanism underlying the second historical permanent host shift may aid in controlling future pandemics of bat RABV and other viruses in terrestrial animals.

RABV has a single, non-segmented negative-strand RNA genome encoding five viral proteins: nucleoprotein (N), phosphoprotein (P), matrix protein (M), glycoprotein (G), and large protein (L)^18^. Of these, G is inserted into the envelope and is responsible for viral attachment to the host cell surface, receptor binding, and membrane fusion^19^. In addition, G also influences viral pathogenicity and neurotropism^20^, inducing the production of virus-neutralizing antibodies^21^. Thus, the G protein clearly plays a key role in determining RABV infection and adaptation to the host. In this study, we dissected the phylogenetic history of the G genes of SCSKV and RRV and found that they are recombinants stemming from the same recombination events. The key genetic component responsible for receptor binding in RRV and SCSKV derives from a carnivore RABV, while the remaining half of the gene derives from a bat RABV. Therefore, the homologous recombination (HR) event appears to have allowed the bat RABV to rapidly adapt to a carnivore host, initiating an epidemic among the carnivores of North America.

## Materials and Methods

### Viruses and sequences

In order to ensure the authenticity of the data, sequences of RRV and SCSKV G genes were collected from several previous reports (n = 148, see Additional File 1)^11, 15, 22–24^ and from reference RABVs from GenBank (n = 450, see Additional File 2) including global RABV lineages. These were aligned using CLUSTALX v1.81^25^. Xia’s test was performed to measure substitution saturation of the sequence alignment^26^. The alignment files for the recombination analysis are available upon request.

### Phylogenetic analysis

RABV phylogenies were reconstructed with the neighbor-joining (NJ), maximum parsimony (MP), and maximum likelihood (ML) methods utilizing MEGA v6^27^. The best nucleotide substitution model was determined using the model selection program implemented in MEGA v6, and the gamma parameter was also estimated for the NJ method. The robustness of each monophyletic group was assessed with the bootstrap method with 1000 replicates. A monophyletic group with >70% bootstrap support was considered a robust lineage. A Shimodaira-Hasegawa test implemented in the Treetest program (http://aix1.uottawa.ca/~sarisbro/) was employed to determine whether the phylogenetic trees were significantly different^28^.

### Recombination analysis

The putative recombinant sequence and its parent sequences were identified using the SimPlot^29^ and RDP 3.0^30^ software packages, as previously reported^31^. Analyses were also carried out with the Bootscan program in SimPlot using the putative recombinant sequence as a query. Mosaicism is suspected when high levels of phylogenetic relatedness are obtained between the query sequence and more than one reference sequence in different genomic regions. Finally, recombination breakpoints were analyzed by maximizing χ^2^, employing a combination of SimPlot and RDP 3.0.

### Convergent evolution analysis

Due to genetic mutations and restrictions on protein function, convergent evolution may alter the topology of a phylogenetic tree inferred from gene sequences. Because adaptive selection for convergent evolution operates mainly at the amino acid sequence level of a protein^32^, it can be identified by comparing phylogenetic trees inferred from nucleotides at the third codon position with those based on first and second codon positions for the gene of interest. Different topologies in the two resulting trees suggests that convergent evolution has occurred during evolutionary history. We reconstructed the phylogenetic histories of the recombinant region of the G gene using third vs. first and second codon positions.

### Estimates of the time to the most recent common ancestor (tMRCA)

The time to the most recent common ancestor (tMRCA) of each lineage was calculated to date the recombination event using the Bayesian Markov chain Monte Carlo (MCMC) method implemented in BEAST (version 1.7.2)^33^. Bayesian MCMC analyses were performed with GTR nucleotide substitution models with invariant sites and a gamma distribution of four rate categories (Γ4). Each Bayesian MCMC analysis was run for 10 million states and sampled every 1,000 states. Posterior probabilities were calculated with a burn-in of 1 million states and checked for convergence using Tracer (version 1.4.1) (http://tree.bio.ed.ac.uk).

## Results

### Nearly half of the G gene of SCSKV and RRV is inherited from a carnivore RABV

Sequences of SCSKV and RRV G genes were collected from several previous reports (n = 148, see Additional File 1) and reference RABVs from GenBank (n = 450, see Additional File 2). As previously reported^6, 14^, phylogenetic analysis suggested that they fell in the bat clade (see Additional File 3A). We also retrieved worldwide RABV G genes from GenBank in order to identify their positions in the RABV phylogenetic tree (see Additional File 3B).

**Figure 1:**
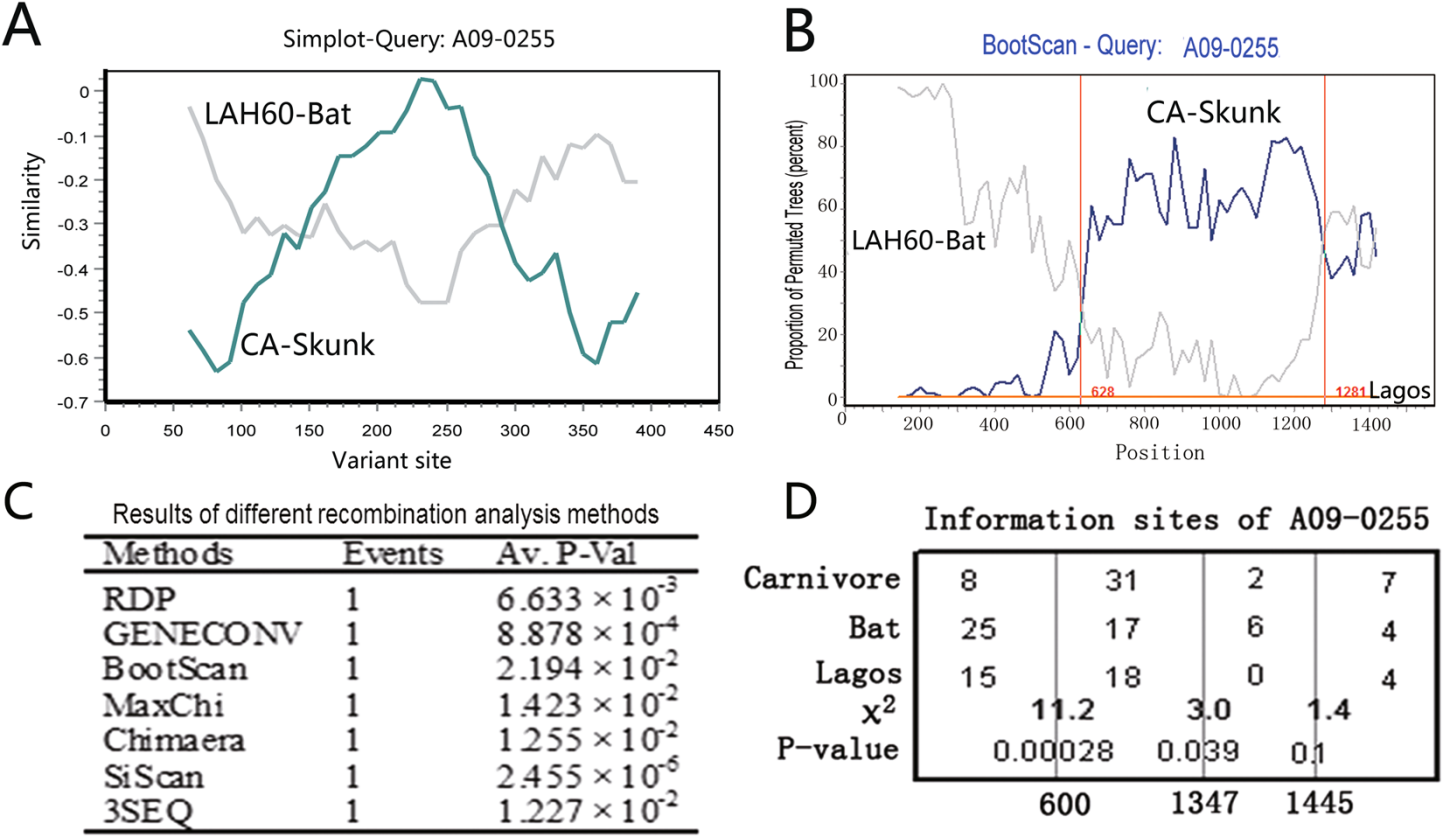
Recombination analysis of SCSKV and bat and skunk RABVs. (**A**) Variable site comparison of the G genes of SCSKV and carnivore and bat RABV representatives, A09-0255, CA, and LAH60. SCSKV A09-0255 was used as a query. The y-axis gives the percent identity between A09-0255 and two representatives of the parental lineages. (**B**) A bootscan plot of A09-0255 with a sliding window of 350 bp and a width of 20 bp using the complete G gene. The y-axis represents the percentage of permuted trees. Two Lagos bat viruses, 8619NGA and 0406SEN, were used as an outgroup. Analyses were performed using Simplot software. Vertical lines represent crossover sites. (**C**) Statistical analysis of the recombination event in the A09-0255 G gene using RDP 3.0. P-values of seven statistical methods implemented in RDP 3.0 to identify recombination are listed. (**D**) Analysis of informative sites for identification of potential recombination breakpoints. Three sites with maximum χ^2^ values are indicated by three vertical lines. The χ^2^ value and P-value of Fisher’s exact test are shown near the vertical lines. The Lagos virus isolate 8619NGA is used as the outgroup.

Next, we further investigated the G gene sequences from three representatives of the SCSKV variant, the carnivore lineage, and the bat lineage: A09-0255, an American wolf isolate belonging to the SCSKV genotype; CA, isolated from a North American skunk of the cosmopolitan clade virus; and LAH60, isolated from a North American bat (*Lasiurus borealis*/*cinereus*), which shares the highest similarity to SCSKV among available bat RABVs. We initially compared all of the variable sites in the A09-255, CA, and LAH60 G genes and discovered two crossover sites. Between the two crossover sites, A09-255 shared higher similarity with the carnivore virus, while outside of this region, A09-255 exhibited higher sequence identity with the bat RABV (Fig. 1A). Comparison of a complete RRV genome with the bat and carnivore RABVs also demonstrated a similar G gene pattern (see Additional File 4). Bootscan analysis of the G gene with an outgroup (Lagos bat viruses 8619NGA and 0406SEN) indicated that this discordant sequence likely derives from HR. Two crossover sites were also identified in the bootscan analysis (Fig. 1B). Between the two crossover sites, A09-255 was found to be homologous to carnivore RABVs, while it was more similar to bat RABVs outside of this region. Taken together, these results suggest that the G gene of SCSKV likely underwent a recombination event, resulting in the two breakpoints. Using the three representative isolates, the recombination event was further examined through seven recombination analysis methods implemented in the RDP software package, which properly allows for statistical analysis of the probability of recombination (Fig. 1C).

To determine the two putative breakpoints, we next analyzed informative sites using Fisher’s exact test (Fig. 1D). Two putative breakpoints were discovered at positions 600 (χ_max_^2^ = 11.2, P < 0.0005) and 1347 (χ_max_^2^ = 3.0, P < 0.05) of the G gene open reading frame (ORF), suggesting that over half of the G gene (747/1575 nucleotides = 47.4% of the SCSKV G gene) may be derived from an American carnivore RABV.

**Figure 2:**
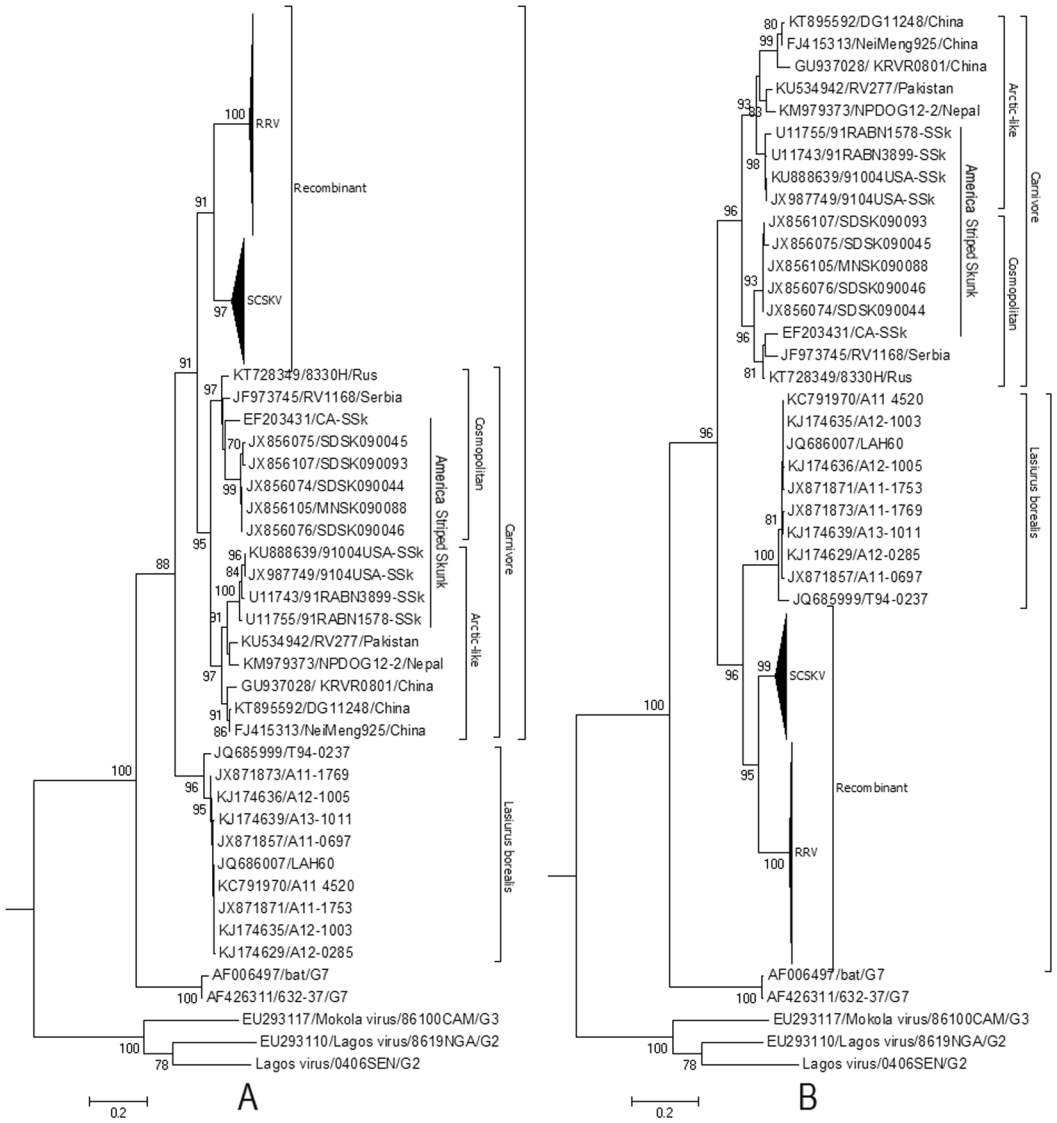
Phylogenetic patterns of the different regions of the G gene delimited by the putative breakpoints. (**A**) Phylogenetic history of the G gene region from positions 601 to 1347. (**B**) Phylogenetic history of G gene regions from positions 1 to 600 and 1347 to 1515 of the alignment. The evolutionary history was inferred using maximum likelihood (ML) methods. Bayesian Information Criterion within MEGA6 was used to find the best nucleotide substitution model for ML analyses. ML trees were constructed using the Tamura 3-parameter nucleotide substitution model. Among-site rate variation was gamma-distributed with invariant sites (G + I) (**A**) or gamma-distributed (G) (**B**) with four rate categories (Γ4). Bootstrap values (>70%) are listed above the branches. Evolutionary analyses were conducted in MEGA v6. G2, G3, and G7 indicate genotypes 2, 3, and 7 of *Lyssavirus*; NCSK, north-central skunk; SCSK, south-central skunk; Rac, raccoon. Cosmopolitan and Arctic-like RABVs are also included.

The gold-standard approach for confirming the presence of recombination is a set of statistically incongruent phylogenetic trees^34^. Inside the breakpoints, they were nested within the clade of carnivore RABVs with 91% (of 1000 replicates) bootstrap support (Fig. 2A). Outside of the two breakpoints, however, SCSKV and RRV clustered into the American bat lineage with 96% (of 1000 replicates) bootstrap support (Fig. 2B). The topologies of these two trees were significantly different (Shimodaira–Hasegawa test, P < 0.0001).

### Convergent evolution does not cause phylogenetic discrepancy of the HR region

**Figure 3:**
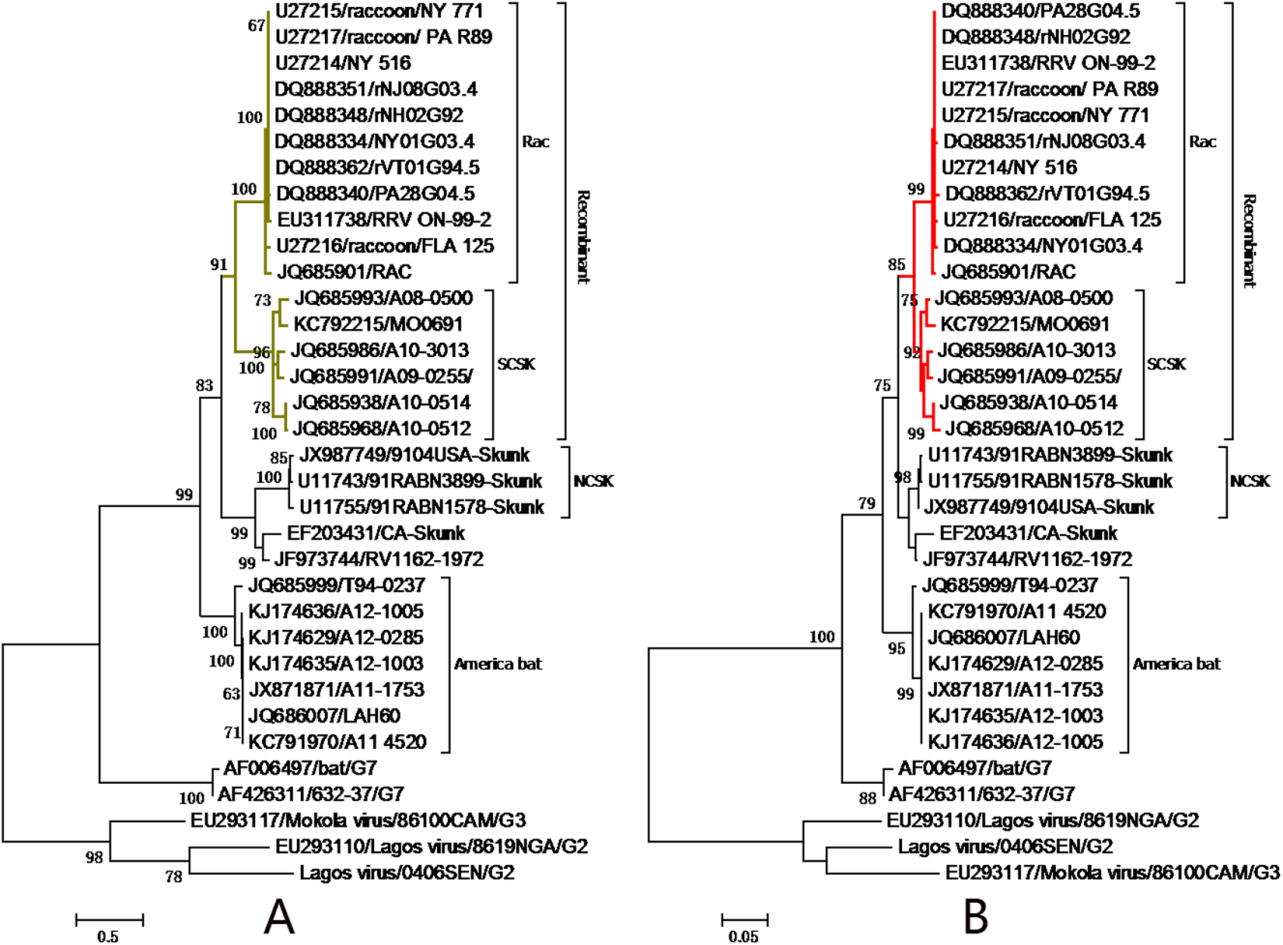
Analysis of convergent evolution in the recombinant region. Phylogenetic histories of the G gene recombinant region inferred from codon position 3 (based on the Kimura 2-parameter + invariable sites models) (**A**) and positions 1 and 2 (based on Tamura 3-parameter + Γ4 models) (**B**). These trees were reconstructed using the ML method implemented in MEGA v6. Please refer to Fig. 4 for detailed descriptions. NCSK, north-central skunk; SCSK, south-central skunk; Rac, raccoon.

To assess whether the significant discrepancy in the G gene’s phylogenetic history may have resulted from convergent evolution, phylogenies of the incongruent region were reconstructed using nucleotides from codon position 3 (Fig. 3A) and from positions 1 and 2 (Fig. 3B). The topologies of the two trees are identical, indicating that HR caused the phylogenetic difference seen in the RRV and SCSKV G gene, rather than convergent evolution.

### The recombination event may have occurred several hundred years ago

**Figure 4:**
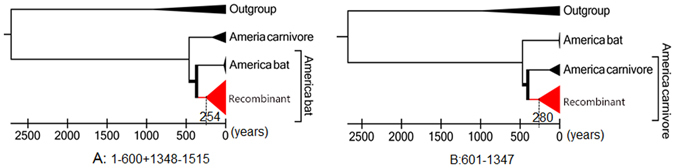
Estimation of the age of the recombination event using Bayesian Markov chain Monte Carlo (MCMC). (**A**) Bayesian tree based on the region outside the two breakpoints. (**B**) Bayesian tree based on the region between the two breakpoints. The scale axis shows the years to 2013. The dotted line indicates the time to the most recent common ancestor (tMRCA) of the recombinant lineage.

Depending on a molecular clock model based on genetic divergence of rabies virus variants in bats of different species, the common ancestor of North American bats lived around 1651 to 1660^35^. To estimate the timing of the recombination event, we also calculated the tMRCA of the recombinant lineage according to each sequence region delimited by the two breakpoints using a Bayesian evolutionary analysis method. The incongruent origins of the recombinant lineage were also reflected in the different topologies of the two Bayesian trees (Fig. 4). The resulting trees estimated that the tMRCA of the recombinant was 254 (95% highest probability density, HPD: 133–491; Fig. 4A) or 280 (95% HPD: 140–650; Fig. 4B) years ago. Thus, the recombination event may have occurred approximately 250 years ago.

### HR resulted in rapid evolution of the bat RABV G protein

Finally, we compared the amino acid sequences of the G proteins of the recombinant lineage and its parent lineages and found 17 positions between the two putative breakpoints where the recombinant is more similar to the carnivore RABV (see Additional File 5). We also assessed d_N_/d_S_ ratios employing Datamonkey^36^ and found no positively selected sites along the stem branch of the recombinant lineage at a 10% significance level. However, in the recombinant sequence of RABV following the host switch, the non-synonymous substitution rate is up to 6% (versus 0.4% in the same region of the virus without recombination and remaining associated with bat host). These results indicate that the recombination event induced rapid changes to the bat RABV G protein.

**Figure 5:**
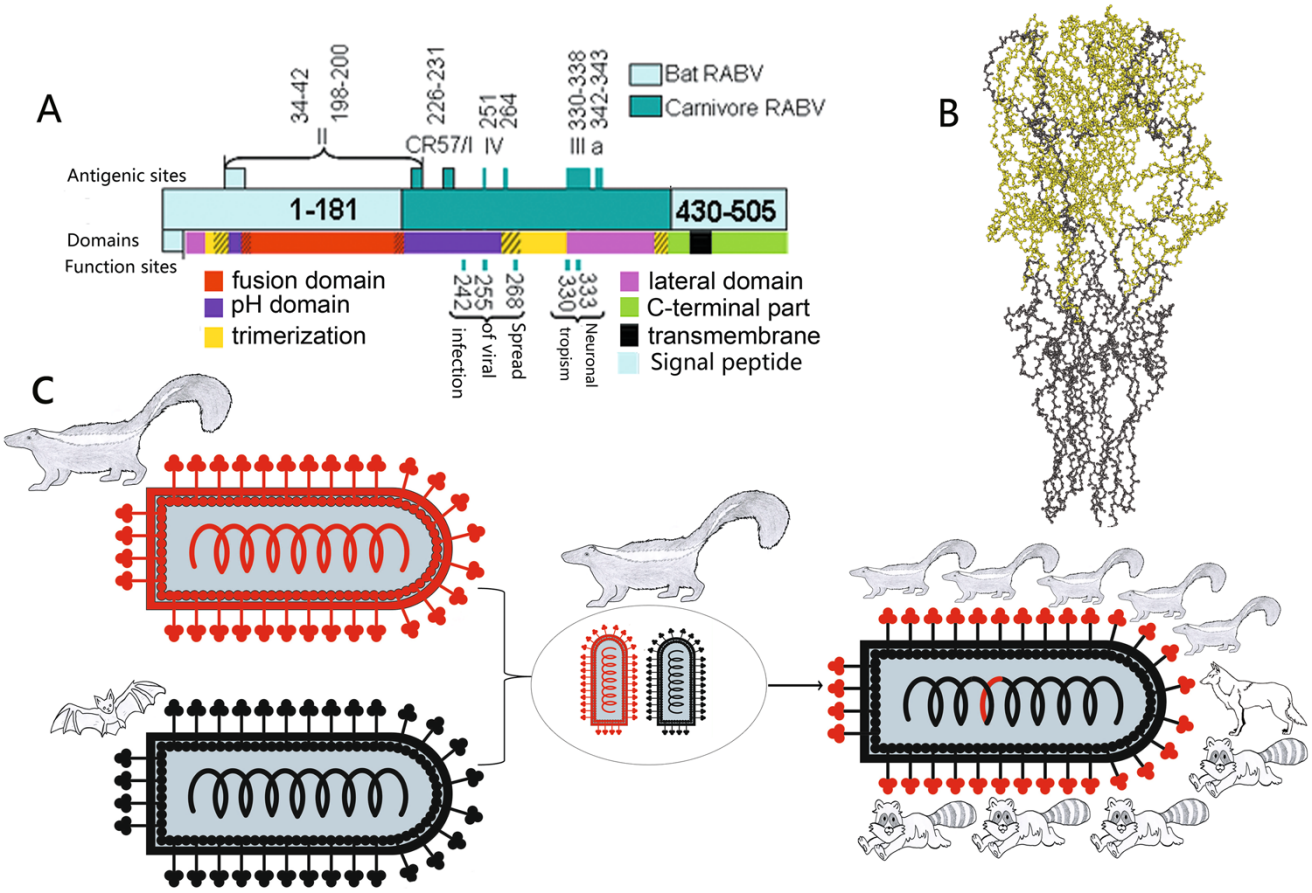
Schematic representation showing the different origins of the recombinant glycoprotein. (**A**) Schematic representation of the mosaic glycoprotein. Light and dark colors indicate regions descended from bat and carnivore RABVs, respectively. Different colors represent the different functional regions of the G protein: lateral domain (domain I), pink; trimerization domain (domain II), yellow; pH domain (domain III), purple; fusion domain (domain IV), red; C-terminal region, green; transmembrane region, black. The main antigenic sites/epitopes and functional sites corresponding to host tropism also are listed. Amino acid numbering is derived from the mature protein minus the signal peptide. (**B**) The ball and stick model of 3D structure of the mosaic trimeric G protein ectodomain. The 3D structure is inferred from that of vesicular stomatitis virus G protein (PDB ID: 5I2M). The amino acid residues obtained from skunk RABV and bat RABV are displayed in bright green and black, respectively. (**C**) Schematic diagram of the origin of the recombinant. On the left, the two parent viruses are shown with red and black denoting regions derived from the skunk and bat RABVs, respectively. In the center, the two viruses come into contact and recombine inside a skunk cell. On the right, the mosaic offspring with a carnivore RABV-derived G-protein head circulates in carnivores.

The recombinant region derived from the carnivore RABV G gene encodes three of the four functional G protein domains: the lateral domain, the trimerization domain, and the pH domain (Fig. 5A). These constitute 84% of the amino acids in the head region of the trimeric G protein hairpin conformation that is exposed on the outside of the virus (Fig. 5B).

## Discussion

We found that the bat virus resulting in the second permanent RABV host shift was derived from an HR event hundreds of years ago between a parental American carnivore strain and a parental bat strain. The bat strain provided most of the mosaic genome except for half of the G gene (see Additional File 4). In the recombinant G gene ORF, the region from positions 601 to 1347 (747 nucleotides) derives from a carnivore RABV, while the rest of the gene derives from the bat RABV. All examined recombinants shared an identical recombination signal in their G genes (Fig. 2), suggesting that these viruses descend from a single ancestor that experienced the recombination event.

We are confident that the sequence data included in this study are reliable enough for recombination analysis. First, all available recombinant family members (>140 isolates) exhibited a similar recombination signal in the G gene, and they were isolated and sequenced by independent research groups, discounting the possibility that the signal derives from artificial PCR or sequencing errors. Secondly, we identified two statistically significant breakpoints in the gene. Finally, sequence identities and phylogenic histories were distinctly different on either side of these apparent breakpoints.

Recombination may be the most economic genetic strategy for altering the host tropism of a virus, allowing a spillover virus to rapidly obtain key genetic material from viruses familiar with the environment of the new host species and thereby reducing the phylogenetic barriers between distant host species. For example, influenza A viruses are notorious for causing frequent pandemics because chicken, pig, and human viral strains can exchange their genomic segments through genetic reassortment, a type of recombination seen in viruses with segmented genomes^37, 38^. In retroviruses, HR between viruses from different primate hosts is associated with the emergence of the human immunodeficiency virus^39^. Recombination has also led to changes in host tropism in positive-strand RNA viruses, such as coronaviruses (CoVs). The CoV that causes severe acute respiratory syndrome (SARS) may have arisen from a recombination event between a bat CoV and another virus before infecting human and carnivore hosts^40^. A Middle East respiratory syndrome (MERS) CoV recombinant lineage has been dominant since December 2014 and subsequently led to human outbreaks in 2015^41^. Despite such examples in other viruses, HR is commonly thought to be rare or absent among negative-strand RNA viruses (NSRVs)^40, 42^. Therefore, recombination has not typically been considered as a genetic mechanism behind NSRV host shifts^40^.

Recombination between RABVs is also controversial. It has been proposed that HR between rabies viruses does not occur^6, 14^. However, we recently isolated two RABV strains with mosaic N genes in China^31^. Interestingly, this study provides robust evidence supporting the fact that HR is an important genetic mechanism driving RABV evolution. Despite this, HR between bat and carnivore RABVs may be uncommon, as it may be rare that the two viruses meet in the same host neuron. This would be consistent with the fact that there have been only two permanent host shifts observed over the long time-span of lyssavirus evolution^6^.

In order for a recombination event to take place, co-infection with the parental viruses is necessary. Spillover infections of RABV can occur in the American bat and terrestrial mammals^43^, providing the opportunity for recombination between bat and carnivore RABVs. The most likely hosts for the recombination event derive from natural RABV vectors, where viruses can persist and undergo vertical transmission, thereby increasing the probability of recombination. To date, these mosaic viruses mainly circulate in striped skunk and raccoon, although they can sporadically be isolated from other species, suggesting that other animals may only represent dead-end hosts. The American bat, striped skunk, and raccoon are the natural reservoir hosts of RABV^44^. Therefore, we propose that the recombination event may have occurred in one of these three species. Moreover, these three RABV natural vectors live within the same environmental niche, increasing the opportunity for co-infection and recombination. Since recombinant offspring have not yet been found circulating in bat, the recombination may have occurred instead in a terrestrial animal infected by the carnivore RABV parent when the bat virus (the major parental strain) infection occurred. Considering that SCSKV is more closely related to the MRCA of the recombinant variant than the raccoon sub-lineage (Fig. 2), an American striped skunk is the most likely candidate host for the co-infection and recombination (Fig. 5C). Future studies should attempt to determine which particular cell types are the most likely host cells for the recombination event in striped skunk.

For a virus to establish a permanent host shift, four stages are necessary: exposure to a new host, infection of the host, onward transmission, and permanent, sustained transmission in the new species^7^. Since bat RABVs are potentially able to infect carnivore species^6, 11, 14, 45^, we also propose that recombination may function as the third and fourth stages of establishment of a permanent host shift by significantly enhancing the adaptability of a bat virus in terrestrial mammals.

The G protein plays a dominant role in the host tropism of RABVs. After binding to a receptor, RABV enters the host cell through the endocytic pathway and subsequently fuses with a cellular membrane within the acidic environment of the endosome^46^. Both receptor recognition and membrane fusion are mediated by the G protein, which is trimeric and forms spikes that protrude from the viral surface^47^. In the mosaic G protein, almost all functional sites directly related to host tropism and infectivity are located in the region derived from the carnivore RABV (positions 181 to 431 of the G protein). For example, neuroinvasiveness and receptor binding is known to be associated with lysine (Lys)^48, 49^ and arginine (Arg)^20, 50^ residues present at positions 330 and 333, respectively. In addition, a recent study suggested that positions 242, 255, and 268 affect the efficiency of cell-to-cell transmission of the viral infection^51^. Interestingly, we found that Arg333 of the carnivore RABV replaced Lys333 of the putative major parental strain, LAH60 (see Additional File 5). These changes may lead the recombinant offspring to be better adapted to the new host species than to bats.

The three-dimensional (3D) structure of the mosaic G protein may allow for a better understanding of the influence of the recombination event on the permanent host shift of the recombinant lineage. Although a 3D structure of a RABV G protein is not available, three of the four major antigenic sites/epitopes (I/CR57, III, and IV) are derived from the American carnivore RABV, reflecting the fact that the recombinant region is exposed on the outside of the G protein 3D structure (Fig. 5A). In addition, the mosaic protein structure can also be estimated by studying the G protein of a bullet-shaped virus family member, vesicular stomatitis virus^47^ (Fig. 5B). Among the four functional domains of the mosaic G protein ectodomain (I, lateral domain; II, trimerization domain; III, pH domain; and IV, fusion domain), the parental bat RABV only provided domain IV (residues 53–172, Fig. 5A), which mainly constitutes the stem of the hairpin conformation of the trimeric G protein (Fig. 5B). In contrast, almost all of domains I, II, and III are derived from the parental carnivore RABV (Fig. 5A), forming 84% of the G protein head (Fig. 5B). These domains are involved in receptor recognition and binding, response to pH changes, and G-protein trimerization^47^. It has been reported that mutations in the pH domain affect the structural transition of the G protein^52^ and therefore may alter the ability of RABV to escape antibody neutralization^53^ and to infect cells^54^. Thus, after acquiring these key genetic elements from skunk RABV, the mosaic offspring could have rapidly adapted to permanent residence in the new host species.

In conclusion, we showed that HR likely resulted in increased genetic diversity and rapid evolution of RABVs. Through this mechanism, a bat RABV strain may have acquired the key genetic information from a carnivore RABV to overcome the phylogenetic barrier between *Chiroptera* and *Carnivora* hosts, inducing its permanent transmission in carnivores.

## Electronic supplementary material

Supplementary information
