# Supplementary material for: A permanent host shift of rabies virus from Chiroptera to Carnivora associated with recombination
Source: Sci Rep. 2017 Mar 21;7:289. doi: 10.1038/s41598-017-00395-2 (PMC5428239; doi:10.1038/s41598-017-00395-2)
Supplement: Supplementary file 1 — Supplementary information [file 41598_2017_395_MOESM1_ESM.doc]

# A permanent host shift of rabies virus from *Chiroptera* to *Carnivora* associated with recombination

Authors: Nai-Zheng Ding ^1^, Dong-Shuai Xu^1^, Yuan-Yuan Sun^1^, Hong-Bin He^1*^, Cheng-Qiang He^1^*

^1^ Shandong Provincial Key Laboratory of Animal Resistance Biology， College of Life Science, Shandong Normal University, Jinan 250014, China

## Additional files

##

## Additional file 3. The phylogenetic classification of raccoon-like viruses

(A) The phylogenetic classification of raccoon-like viruses (B) and their phylogenetic position in global RABVs. Based on the G gene, the evolutionary history was inferred using the Neighbor-Joining (NJ) method. The percentage of replicate trees in which the associated taxa clustered together in the bootstrap test is shown above the branches. The tree is drawn to scale, with branch lengths in the same units as those of the evolutionary distances used to infer the phylogenetic tree. The evolutionary distances were computed using the Maximum Composite Likelihood method and are presented as the number of base substitutions per site. Evolutionary analyses were conducted in MEGA5. MP, monophyletic group; TMA, terrestrial mammal animals.


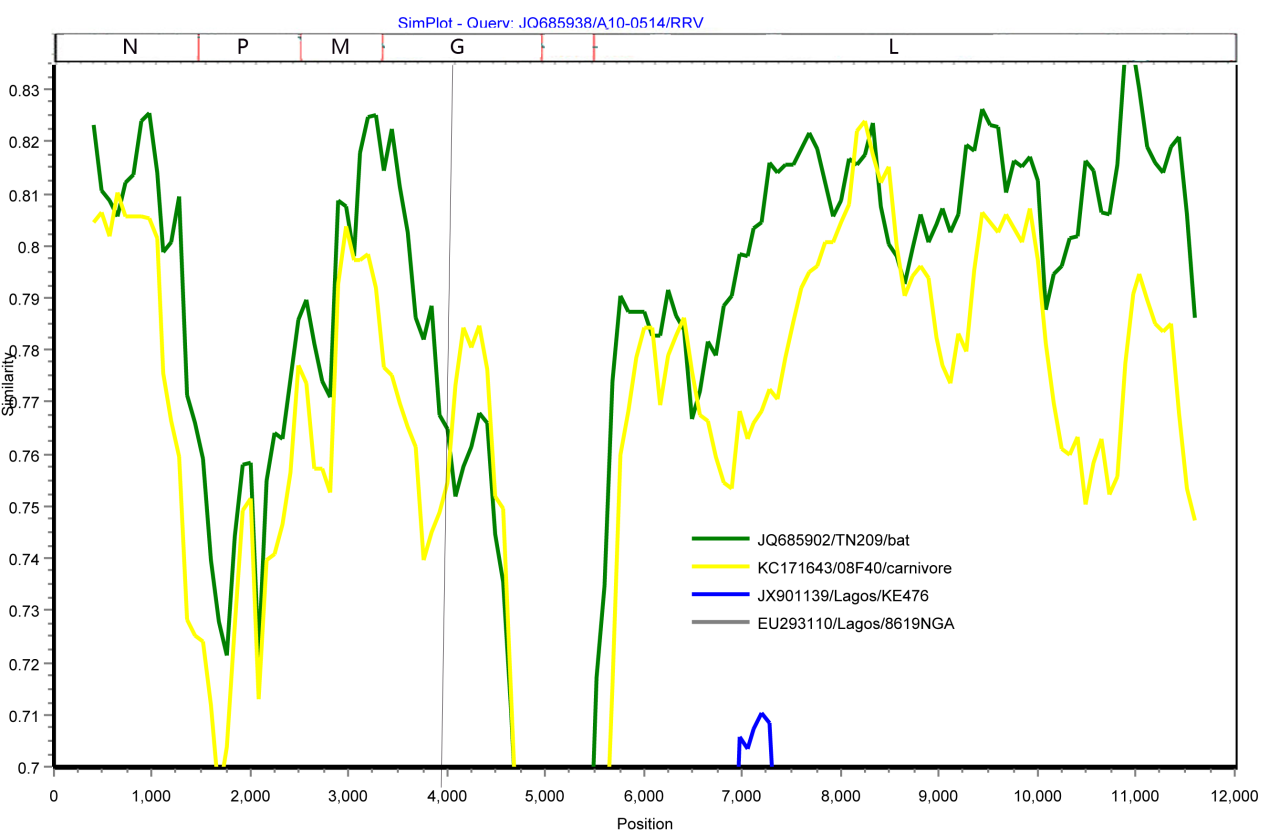


**Additional file 4. Sequence comparison of RRV, bat RABV, carnivore RABV and Lagos virus genome.**

Complete genomes of several representatives of RRV, bat RABV, carnivore RABV and Lagos virus were compared. RRV was used as the query. Other virus was respectively shown with different colors. The y-axis shows the percentage of similarity between RRV and other viruses respectively.


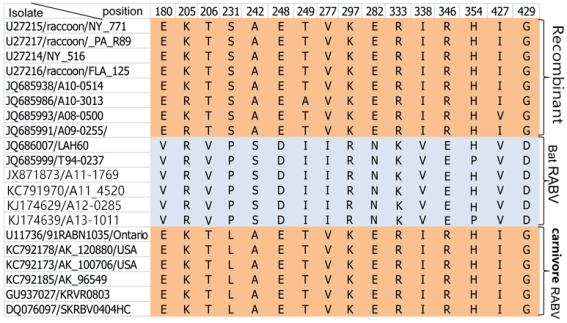


**Additional file 5. Amino acid (AA) comparison of RRV and its parents in the recombination region.**

18 variant sites of recombination region were listed to suggest that RRV has more similar to carnivore RABV. Representatives of each virus lineage were shown with identical color. Carnivore and bat RABV lineages were indicated with different colors respectively. Orange represents carnivore RABV; light blue indicates bat RABV.
